# The performance of detecting *Mycobacterium tuberculosis complex* in lung biopsy tissue by metagenomic next-generation sequencing

**DOI:** 10.1186/s12890-022-02079-8

**Published:** 2022-07-28

**Authors:** Meng Fu, Le-Jie Cao, Huai-Ling Xia, Zi-Mei Ji, Na-Na Hu, Zai-Jun Leng, Wang Xie, Yuan Fang, Jun-Qiang Zhang, Da-Qing Xia

**Affiliations:** 1 Department of Respiratory and Critical Care Medicine, The First Affiliated Hospital of USTC, Division of Life Science and Medicine, University of Science and Technology of China, No. 17 Lujiang Road, Hefei, 230001 Anhui China; 2 Anhui Province Key Laboratory of Medical Physics and Technology, Institute of Health and Medical Technology, Hefei Institutes of Physical Science, Chinese Academy of Sciences, Hefei, 230031 Anhui China; 3 University of Science and Technology of China, Hefei, 230026 Anhui China

**Keywords:** *Mycobacterium tuberculosis complex*, Diagnosis, Pulmonary tuberculosis, Metagenomic next-generation sequencing, mNGS

## Abstract

**Background:**

Tuberculosis (TB) is a chronic infectious disease caused by the *Mycobacterium tuberculosis complex* (MTBC), which is the leading cause of death from infectious diseases. The rapid and accurate microbiological detection of the MTBC is crucial for the diagnosis and treatment of TB. Metagenomic next-generation sequencing (mNGS) has been shown to be a promising and satisfying application of detection in infectious diseases. However, relevant research about the difference in MTBC detection by mNGS between bronchoalveolar lavage fluid (BALF) and lung biopsy tissue specimens remains scarce.

**Methods:**

We used mNGS to detect pathogens in BALF and lung biopsy tissue obtained by CT-guide percutaneous lung puncture (CPLP) or radial endobronchial ultrasound transbronchial lung biopsy (R-EBUS-TBLB) from 443 hospitalized patients in mainland China suspected of pulmonary infections between May 1, 2019 and October 31, 2021. Aim to evaluate the diagnostic performance of mNGS for detecting MTBC and explore differences in the microbial composition in the 2 specimen types.

**Results:**

Among the 443 patients, 46 patients finally were diagnosed with TB, of which 36 patients were detected as MTBC positive by mNGS (8.93%). Striking differences were noticed in the higher detection efficiency of lung biopsy tissue compared with BALF (*P* = 0.004). There were no significant differences between the 2 specimen types in the relative abundance among the 27 pathogens detected by mNGS from the 36 patients.

**Conclusions:**

This study demonstrates that mNGS could offer an effective detection method of MTBC in BALF or lung tissue biopsy samples in patients suspected of TB infections. When it comes to the situations that BALF samples have limited value to catch pathogens for special lesion sites or the patients have contraindications to bronchoalveolar lavage (BAL) procedures, lung biopsy tissue is an optional specimen for MTBC detection by mNGS. However, whether lung tissue-mNGS is superior to BALF-mNGS in patients with MTBC infection requires further prospective multicenter randomized controlled studies with more cases.

## Background

Infectious diseases remain the leading cause of morbidity and mortality around the world [1].The lung infection, the most common infectious disease, has attracted wide concern, especially when it occurs in the form of explosive emerging infectious diseases such as severe acute respiratory syndrome (SARS) and coronavirus disease 2019 (COVID-19) [2, 3]. However, some specific pulmonary infectious diseases should not be ignored. Pulmonary tuberculosis (PTB), a chronic infectious disease caused by the *Mycobacterium tuberculosis complex* (MTBC), remains a major global public health problem that affects at least a quarter of the world’s population each year and causes high morbidity and mortality worldwide, particularly in developing countries, such as India, China and South Africa and so on [4, 5].

The MTBCs can invade multiple systems and organs, such as the lymph nodes, colon, liver, and spine, with lung infection being the most common form [6]. Active patients with PTB, the main source of infection, usually expel bacteria by talking, coughing, or sneezing, spreading the pathogen through airborne particles inhaled by others [7]. The accurate and early detection of MTBC is of great significance for the clinical diagnosis and treatment of PTB, and ultimately helps reduce the spread of PTB. The rapid development of metagenomic next-generation sequencing (mNGS) recently has shown promising and satisfying applications in medical microbiology [8]. mNGS may be particularly useful for samples from a sterile site, such as those from BALF, cerebrospinal fluid, blood, or biopsy tissue. BALF is considered a sterile type of specimen that is suitable for detecting pathogens of respiratory infections. Meanwhile, lung biopsy tissue specimens, the gold standard of diagnosis of cancer, gradually being used to comprehensively detect pathogens of infectious diseases. However, for patients with peripheral pulmonary lesions (PPLs) or who have contraindications of bronchoalveolar lavage (BAL) procedures, the role of BALF in MTBC diagnosis is limited. Lung biopsy specimens may be a good alternative to BALF for MTBC detection under the abovementioned circumstances. Up to now, relevant research about the difference in MTBC detection by mNGS between BALF and lung biopsy tissue specimens remains scarce.

This study comprised 2 parts. We first evaluated the diagnostic performance of mNGS for detecting MTBC between lung biopsy tissue and BALF. The lung biopsy tissue was obtained by radial endobronchial ultrasound transbronchial lung biopsy (R-EBUS-TBLB) or CT-guide percutaneous lung puncture (CPLP). And then we explore differences in the microbial composition in the 2 specimen types to analyze possible relationships between MTBC and other pathogens.

## Methods

### Study design and subjects

**Fig. 1:**
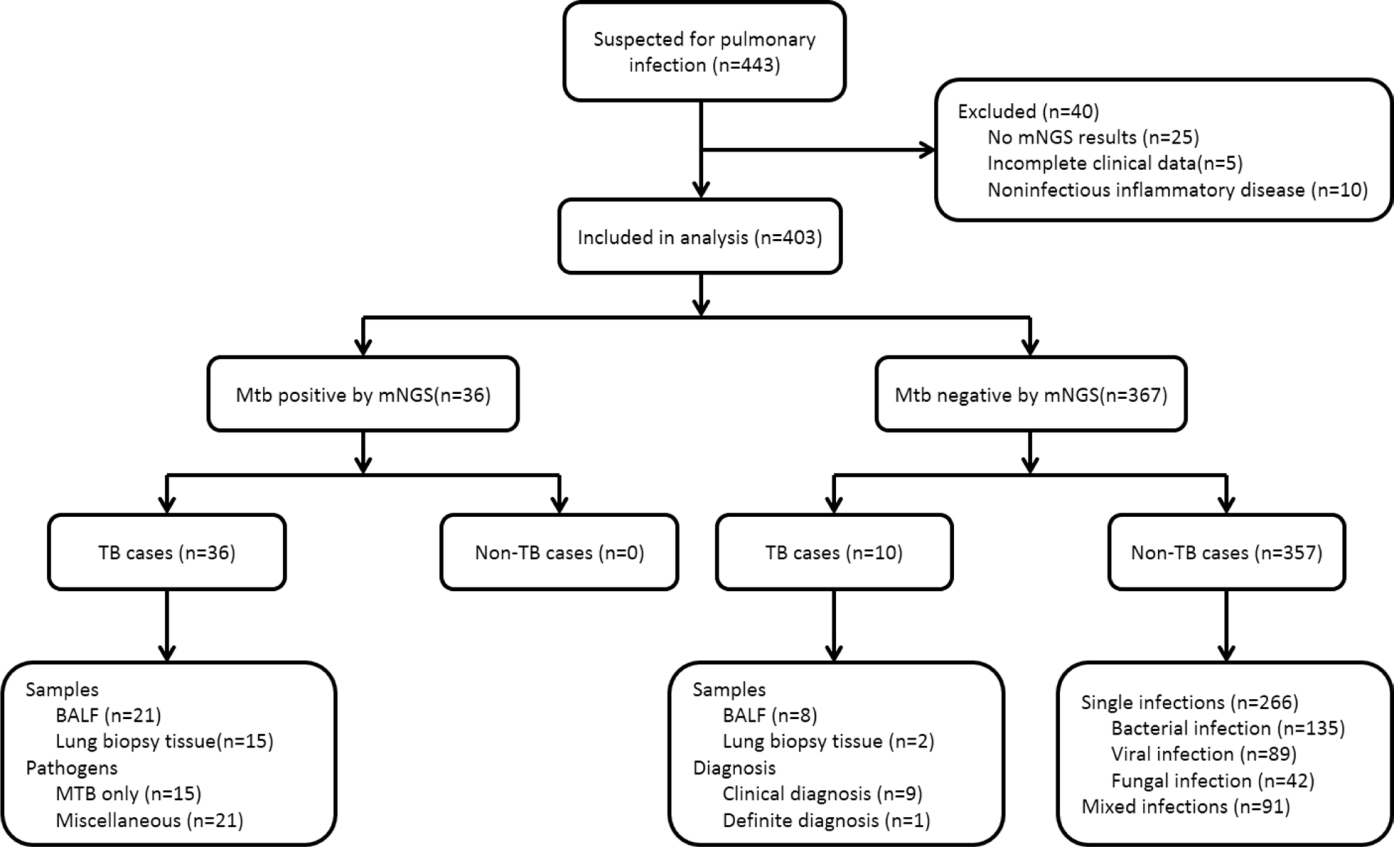
Study design

We retrospectively analyzed the medical records of 443 hospitalized patients who were clinically and radiologically suspected of having pulmonary infection at The First Affiliated Hospital of University of Science and Technology of China (Anhui Provincial Hospital), a provincial tertiary first-class general hospital, in mainland China from May 1, 2019 and October 31, 2021. We excluded patients who met one of the following 4 criteria: (1) no mNGS results, (2) finally were diagnosed with non-infective diseases, (3) incomplete clinical data, (4) coinfection with HIV (Fig. 1). The standard of diagnosis refers to the WHO consolidated guidelines on tuberculosis 2021 version [9]. The patients detected as MTBC positive by mNGS were enrolled in the statistical analysis of the study. Demographic characteristics and clinical information were gathered by electronic medical records, including gender, age, smoking habit, family history, disease history, previous anti-TB/antibacterial therapy, computerized tomography (CT) diagnosis, Sputum Stain, GeneXpert MTB/RIF (Xpert).

### Standard of diagnosis of PTB

The diagnostic criteria for PTB were in accordance with the following several aspects including (1) Clinical diagnosed PTB referred to patients with typical chest radiograph findings and met any of the following: typical clinical presentation of PTB or positive purified protein derivative (PPD) test. Relevant symptoms and radiographic lesions were improved after anti-TB treatment, even if they had negative findings. (2) Definite PTB referred to patients with positive cultures or nucleic acid for MTBC detection in sputum or BALF; patients with at least 1 positive sputum smear; patients with positive pathologic results indicative of PTB; patients with positive Xpert assay results [9].

### Ethics statement and specimen collection

All study methods were carried out in accordance with the Declaration of Helsinki. Ethical approval was achieved from the ethics committee of the First Affiliated Hospital of University of Science and Technology of China (Anhui Provincial Hospital), and written informed consent was obtained from each patient before performing bronchoscopy or lung biopsy. The samples of patients enrolled in our study included BALF and lung biopsy tissue. Bronchoscopy with BAL is often indicated for the definitive diagnosis of PTB, especially when empiric anti-TB treatments fail, and its safety and tolerability profiles have already been confirmed in previous clinical operations [10]. Lung biopsy tissue was obtained from CPLP or R-EBUS-TBLB. Our respiratory endoscopy and intervention center has developed standardized procedures for specimen collection (1) All patients were examined by electronic bronchoscopy in our hospital, and chest CT showed that the lesions were brushed, followed by BAL, (2) The brush specimen of bronchoscopy was routinely tested by acid fast bacillus (AFB) smear and cytopathology, (3) BALF and lung biopsy tissue was preserved in a specialized refrigerator at −80 °C before mNGS test and all specimens were sent for mNGS within 1 h.

### Sample preparation and DNA extraction

The total volume rang 10 to 12 mL BALF sample from patients was collected according to a standard procedure, of which 1.5 ml precipitation was extracted by centrifugation after inactivation were directly collected in deoxyribonuclease (DNase)-free tubes for the identification of potential pathogens. Lung biopsy tissue required a special procedure before the nucleic acid extraction: the tissue sample was ground into homogenate before deoxyribonucleic acid (DNA) extraction. All samples were stored in a refrigerator at −80 °C before the test. Samples were prepared for wall-breaking reaction, and 200 ng DNA was extracted by the TIANamp Micro DNA Kit (DP316, Tiangen Biotech).

### Construction of DNA libraries and sequencing

Our hospital cooperated with the Beijing Genomics Institute (BGI) to establish a localized mNGS laboratory in our hospital for rapid and high-quality etiological detection. The physicians in the department of clinical laboratory of our hospital used MGISEQ-2000 platform, providing by BGI, to conduct mNGS detection and interpretation of reports. The extracted DNA was sonicated and 200–300 bp DNA fragments were obtained. Then, DNA libraries were constructed through DNA-fragmentation, end-repair, adapter-ligation, and polymerase chain reaction (PCR) amplification. Qualified libraries were pooled, DNA Nanoball (DNB) was made using single-stranded DNA circles, and sequenced by MGISEQ-2000 platform.

### Bioinformatic analysis

High-quality sequencing data were calculated by removing low-quality reads, followed by subtracting the human host sequence of the human reference genome (hg38) using Burrows-Wheeler alignment (ftp://ftp.ncbi.nlm.nih.gov/genome). The remaining sequence data were aligned to the Microbial Genome Database. The database used for this study contains 2,328 bacteria, 199 fungi, 4,189 viruses, 135 parasites, 83 mycobacteria, and 41 mycoplasma/Chlamydia, which were associated with human diseases.

### Criteria for a positive mNGS result

1. Bacteria (mycobacteria excluded), viruses and parasites: mNGS identified a microbe whose coverage rate scored tenfold higher than that of any other microbe [11].
2. Fungi: mNGS identified a microbe whose coverage rate scored fivefold higher than that of any other fungus because of its low biomass in DNA extraction [12, 13].
3. Mycobacteria: MTBC was considered positive when no less than 1 read was mapped to either the species or genus level because of the low yields of DNA extraction and rare specimen contamination [14, 15]. Nontuberculous mycobacteria (NTM) were identified as positive when the mapping read number (genus or species level) was in the top 10 in the bacterial list due to the balance of hospital-to-laboratory environmental contamination [16] and low yield rate [17].

### Statistical analysis

The sensitivity and specificity of different ways of obtaining samples were analyzed. 95% confidence intervals (CI) are displayed in the results figures. Comparative analysis was conducted by Pearson χ2 test, Fisher exact test, the Wilcoxon rank sum test or the McNemar test for discrete variables. Statistical analyses and figures were conducted using the SPSS statistical package 24.0 software and GraphPad Prism 9 software. A heatmap and Stacked Bar Chart was drawn using the R language. P-values < 0.05 were considered statistically significant.

## Results

### Patient and sample characteristics

Among the 443 patients suspected of pulmonary infection between May 1, 2019 and October 31, 2021, according to the inclusion and exclusion criteria, 40 patients were excluded. From them, twenty-five patients without mNGS result and five patients without complete clinical data were excluded. Ten patients were not enrolled on account of the final diagnosis of noninfectious inflammatory disease. Four hundred and three patients underwent mNGS tests on direct clinical samples, including BALF or lung biopsy tissue. A total of 36 patients (36/403, 8.93%) were detected MTBC positive, ranging in age 16 to 84 years old, with an age median of 56 years, an average age of 53.3 years (95% CI, 46.2–60.4 years), and the male/female ratio of 13/23. The positive detections from two different sample detection modes were 21 cases in BALF sample, and 15 cases in lung biopsy tissues. Combine with the results of Xpert, treatment, and follow-up, the 36 patients were finally diagnosed with PTB. The clinical and demographic characteristics of the study patients are summarized in Table 1. Among 36 patients detected MTBC positive by mNGS, 26 patients underwent tuberculous testing with mNGS and Xpert simultaneously, and 10 patients carried on mNGS and Xpert, respectively, as part of the validation process. The remaining 367 patients had MTBC-negative results by mNGS detection, including 9 cases diagnosed with clinical PTB and 1 case that were diagnosed with definite PTB for positive Xpert results.

**Table 1:** Clinical information of the enrolled patients with TB

Patient	Gender	Age	Smoking	Disease history	CT diagnosis*	Sample	Sputum stain*	Xpert*
1	M	48	N	N	U/U, M	BALF	Neg × 3	L
2	M	80	1200	Pulmonary emphysema	U/U, M	BALF	Neg × 3	L
3	M	35	N	Uremia, Post renal transplant	U, L/U, M, L	BALF	Neg × 2	L
4	M	29	N	N	U/ (−)	BALF	Neg × 2	M
5	M	62	N	T2DM	U/U	BALF	Neg × 4	L
6	F	19	N	N	L/ (−)	BALF	Neg × 3	L
7	M	68	N	N	U/U, M	BALF	Neg × 3	Neg
8	M	68	1000	Hypertension, LA, Syphilis	L/ (−)	BALF	1 +	H
9	F	77	N	Hypertension	U, L/U, M, L	BALF	Neg × 3	L
10	F	56	N	Hepatitis B	(−)/U, M	BALF	Neg × 2	L
11	F	78	N	COPD, Bronchiectasis, CI	L/M, L	BALF	Neg × 3	M
12	F	78	N	N	L/ (−)	BALF	Neg × 2	L
13	F	45	N	Heart failure, PAH	U/U, M, L	BALF	Neg × 3	M
14	F	30	N	AML, UCBCT	U, L/U, M, L	BALF	Neg × 3	L
15	M	57	N	LUSC of left lung	U/ (−)	BALF	Neg × 2	L
16	M	57	800	N	U, L/U, M, L	BALF	Neg × 2	M
17	M	38	N	N	(−)/U, M	BALF	Neg × 2	L
18	F	24	N	N	U, L/ (−)	BALF	Neg × 3	H
19	M	62	N	N	(−)/U, M	BALF	Neg × 3	H
20	M	35	N	N	(−)/U, M, L	BALF	Neg × 2	L
21	M	81	800	COPD, CHD	(−)/M, L	BALF	Neg × 3	L
22	F	78	N	N	U/ (−)	R-EBUS-TBLB	Neg × 2	H
23	M	38	N	N	U, L/ (−)	R-EBUS-TBLB	Neg × 2	L
24	M	80	2400	N	U/U	R-EBUS-TBLB	Neg × 2	M
25	F	79	N	N	(−)/M, L	R-EBUS-TBLB	Neg × 2	L
26	M	56	N	CHD	U/ (−)	R-EBUS-TBLB	Neg × 3	Neg
27	M	21	N	N	(−)/L	R-EBUS-TBLB	Neg × 2	M
28	F	65	N	N	U, L/U, M, L	CPLP	Neg × 2	L
29	M	32	30	N	(−)/U	CPLP	Neg × 2	M
30	M	25	100	N	U/ (−)	CPLP	Neg × 2	L
31	M	84	600	T2DM, Hypertension	(−)/U	CPLP	Neg × 2	L
32	M	56	N	Esophagus cancer	U/ (−)	CPLP	Neg × 2	L
33	M	55	600	T2DM	(−)/U	CPLP	Neg × 2	H
34	M	41	N	Epilepsy	U, L/U, M, L	CPLP	Neg × 3	L
35	F	16	N	Nephrotic syndrome	(−)/L	CPLP	Neg × 2	L
36	F	67	N	N	L/L	CPLP	Neg × 2	L

*In the column of Smoking and Disease history, the numbers in the table indicate smoking index. The average root number per day multiplied by smoking years of smoking, that is, smoking index. N refers to non-smoking. *In the column of CT diagnosis shows the location of the TB lesion in each patient. The left side of the ‘/’ refers to the left lung and the right side refers to the right lung. U, M, L refer to the upper, middle and lower lobi of lungs, respectively, and ‘−’ refers to lesion-free lungs. *In the column in Sputum stain shows the result and time of sputum stain test. ‘−’ refers to negative. *In the column Xpert, L, M and H respectively refers to low abundance, medium abundance and high abundant. T2DM = Type 2 diabetes, LA = lung adenocarcinoma, COPD = chronic obstructive pulmonary disease, CI = cardiac insufficiency, PAH = pulmonary arterial hypertension, AML = acute myelogenous leukemia, UCBCT = umbilical cord blood stem cell transplantation, LUSC = lung squamous cell carcinoma, CHD = coronary heart disease, R-EBUS-TBLB = radial endobronchial ultrasound transbronchial lung biopsy, CPLP = CT-guide percutaneous lung puncture biopsy

In this study, the 36 mNGS MTBC-positive patients were enrolled and categorized into two groups. Demographic characteristics were collected and analyzed. The average age was 53.7 years (95% CI, 44.6–62.8 years) old among the BALF group. Among them, male patients accounted for 61.9%. The proportion of smoking in the BALF group was 19%. We found that no significant differences in age, male, family history, disease history, and abundance of Xpert and sputum stain were observed between the BALF group and lung biopsy tissue groups (Table 2). Besides, the pathogens were analyzed among all subjects. As shown in Table 2, we found that the patients only detected MTBC in the lung biopsy tissue group significantly more than that in the BALF group (Fisher's exact test, *P* = 0.006).

**Table 2:** Baseline characteristics and pathogens of the study population

	BALF (n = 21)	Lung biopsy tissue (n = 15)	P
Baseline characteristics			
Age (mean), years	53.7	52.9	–
Sex—male, n (%)	13 (61.9)	5 (33.3)	0.176
Smoke, n (%)	4 (19.0)	5 (33.3)	0.443
Family history	0 (0)	0 (0)	–
Disease history	11 (52.4)	6 (40.0)	0.516
ATT before sample collection, n	0	0	–
Pathogen			0.006
MTBC only	4	10	
Miscellaneous	17	5	
Xpert			0.995
High abundant	3	2	
Medium abundance	4	3	
Low abundance	13	9	
Negative	1	1	
Sputum stain			–
Positive	1	0	
Negative	20	0	

### Diagnostic performance of mNGS and Xpert tests for MTBC detection

Then, we focused on assessing the diagnostic performance for mNGS, Xpert, and traditional methods compared with the clinical final diagnosis. We noticed that two of the mNGS-positive cases presented a negative result Xpert. On account of high reads of MTBC (*n* = 1484 and 609) and typical clinical symptoms and imageology characteristics in this case, we gave diagnosis as PTB and observed effective anti-TB treatment in follow-up. Among these 10 patients finally diagnosed as PTB with negative mNGS results, 9 cases with clinically diagnosed PTB were negative for both Xpert and mNGS while 1 case was positive for Xpert. This Xpert-positive case was collected from a BALF sample (Fig. 1). The 10 patients were already put on anti-TB treatment with improved tuberculosis symptoms.

**Fig. 2:**
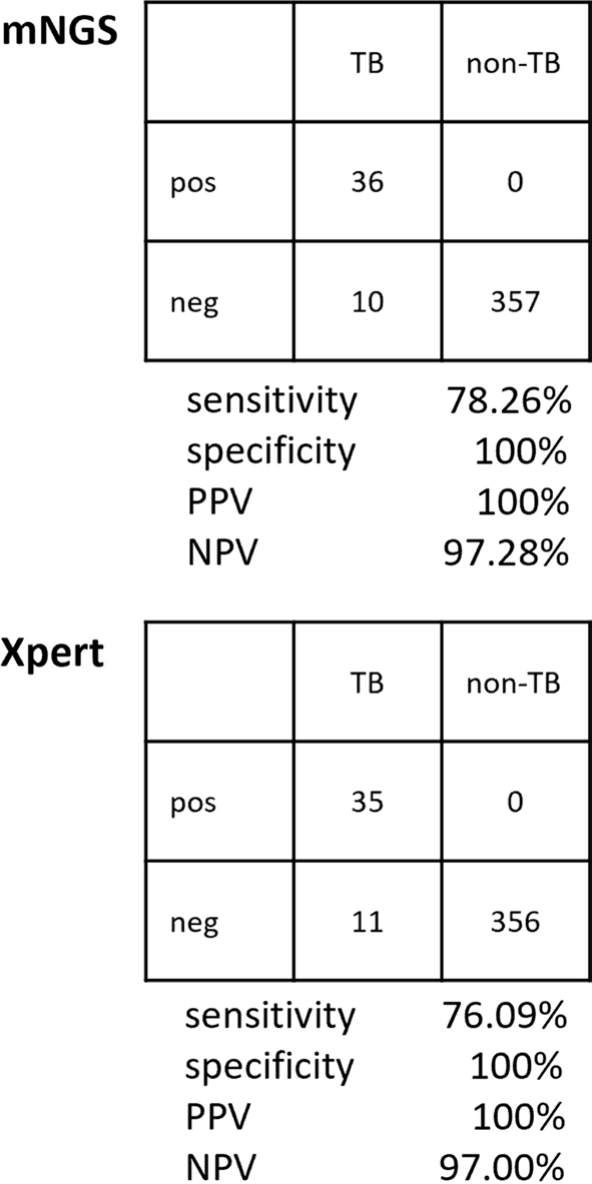
Diagnostic performance of mNGS and Xpert tests for MTBC detection

The sensitivity and specificity of MTBC detection by mNGS was 78.26% (36/46, 95% CI, 65.88%–90.65%) and 100% (36/36) respectively. The positive predictive value (PPV) and negative predictive value (NPV) of MTBC detection by mNGS was 100% (36/36) and 97.28% (357/367, 95% CI, 95.60–98.95%). The sensitivity and specificity of MTBC detection by Xpert was 76.09% (35/46, 95% CI, 63.28–88.89%) and 100% (35/35) respectively. The PPV and NPV of MTBC detection by Xpert was 100% (35/35) and 97.00% (356/366, 95% CI, 95.25–98.76%) (Fig. 2). Overall, mNGS and Xpert presented similar overall MTBC detection efficiency, which was also superior to AFB (2.17%) (Fisher's exact test, *P* < 0.05).

### Comparison of diagnostic performance for MTBC between BALF and lung biopsy tissue

**Fig. 3:**
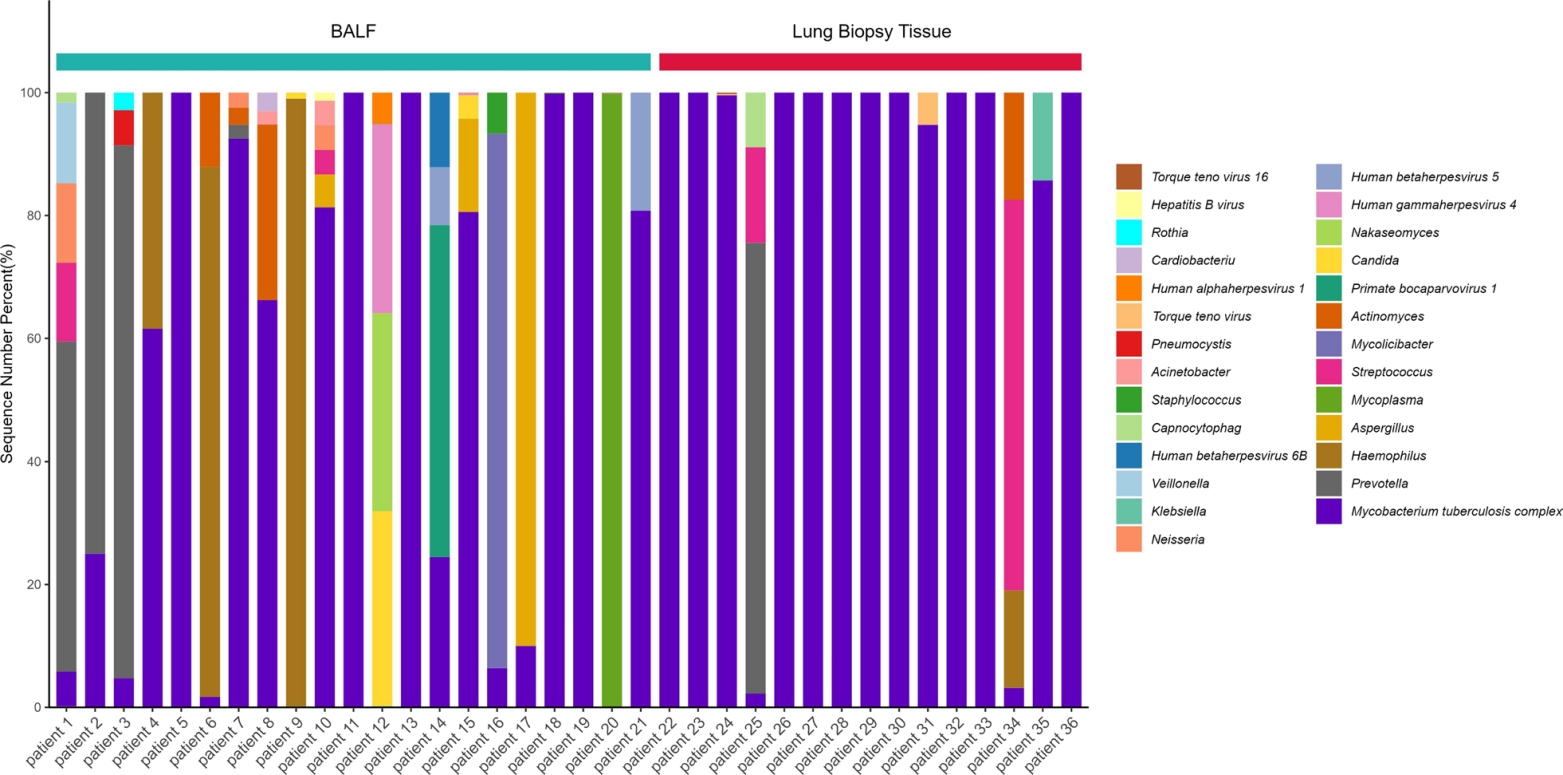
BALF comparison in lung biopsy tissue samples by mNGS from MTBC-positive patients. The stacked histogram were plotted with the percentage of pathogen reads detected by mNGS in each patient's BALF or lung biopsy tissue samples. Differential abundance analysis indicated that the ratio of MTBC reads in the lung biopsy tissue group was significantly higher than that in the BALF group (non-parametric tests, *P* = 0.004). mNGS = metagenomic next-generation sequencing, BALF = bronchoalveolar lavage fluid

A comparison of the relative abundances of genera between the BALF and lung biopsy tissue groups allowed us to identify differences in the diagnostic performance for mNGS in detecting MTBC (Table 3). We counted the proportion of MTBC reads in total sequences of each patient, respectively, it indicated that mNGS possess a higher efficiency in lung biopsy tissue (non-parametric tests, *P* = 0.004) (Fig. 3).

**Table 3:** The detection ratio of MTBC sequence reads

Patient	Sample	MTBC	Total sequences	Detection ratio (%)
1	BALF	656	11,321	5.79
2	BALF	1	4	25
3	BALF	5	105	4.76
4	BALF	578	939	61.55
5	BALF	8	8	100
6	BALF	1	58	1.72
7	BALF	1484	1604	92.52
8	BALF	243	367	66.21
9	BALF	3	1941	0.15
10	BALF	61	75	81.33
11	BALF	16	16	100
12	BALF	74	34,074	0.22
13	BALF	826	826	100
14	BALF	117	478	24.48
15	BALF	61,407	76,219	80.57
16	BALF	22	345	6.38
17	BALF	1	10	10
18	BALF	3231	3236	99.85
19	BALF	2979	2979	100
20	BALF	4	7779	0.05
21	BALF	21	26	80.77
22	Biopsy	6018	6018	99.57
23	Biopsy	11	11	100
24	Biopsy	1611	1618	99.57
25	Biopsy	45	45	2.22
26	Biopsy	609	609	100
27	Biopsy	1	1	100
28	Biopsy	1	1	100
29	Biopsy	757	757	100
30	Biopsy	115	115	100
31	Biopsy	229	229	94.76
32	Biopsy	41	41	100
33	Biopsy	830	830	100
34	Biopsy	63	63	3.17
35	Biopsy	7	7	85.71
36	Biopsy	30	30	100

### Characteristics of microbial composition detected in different types of specimens

**Fig. 4:**
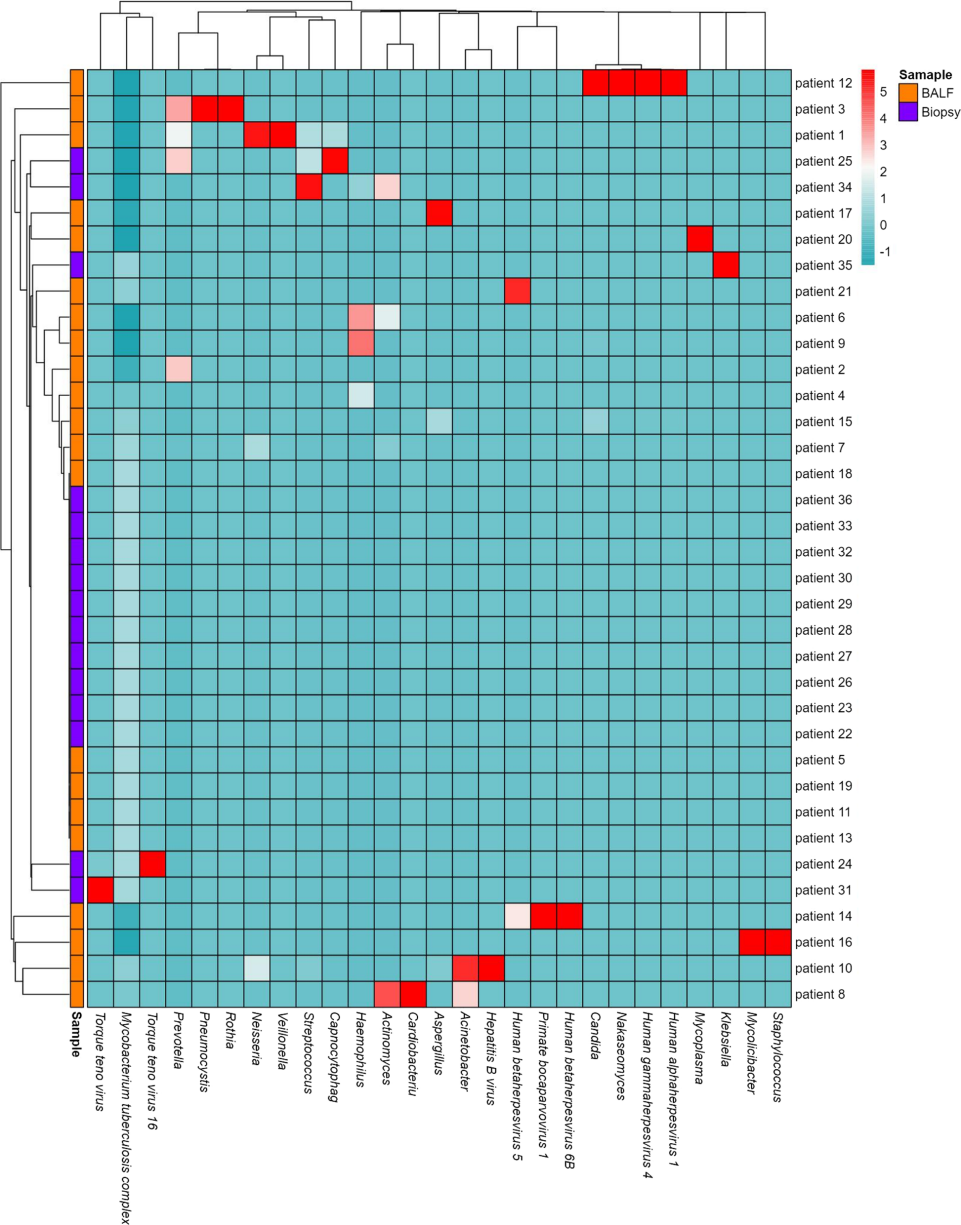
Analysis of the pathogens detected by mNGS. The clustering heatmap, presenting all pathogens, was drawn using the z-scores of reads detected by mNGS

An average of 69,472,264 reads per sample from patients was obtained. Before the analysis of relative abundance, we remove anthropogenic sequences and colonized bacterial sequence reads by comparing to microbial database. To describe the detailed composition features of the microbiota in detail, patients were sorted into two categories as BALF and biopsy groups. The grouped clustering heatmap of pathogen abundances across samples was presented after Z-score was executed to standardize the reads (Fig. 4). It displays the abundance of each category and the relationships between categories and specimens.

In 36 cases detected MTBC positive by mNGS, 14 cases (14/36, 38.89%) were detected MTBC only, while mNGS were also positive for other pathogens in 22 cases (22/36, 61.11%). The species and relative abundance of pathogens detected in enrolled cases were listed in Table 4, respectively. The mNGS sequences detected reads of MTBC ranged from 1 to 61,407, and reads of the other pathogens ranged from 1 to 11,583. After analyzing the relative abundance, we focused on the genera with relative abundance equal to or greater than 0.1%. The genera from top to bottom were *Candida* (8.9871%)*, Aspergillus* (7.5898%), *Nakaseomyces* (7.1741%), *Human gammaherpesvirus 4* (7.0210%), *Mycoplasma* (5.0869%), *Prevotella* (4.0848%), *Haemophilus* (1.5316%), *Human alphaherpesvirus 1* (1.1677%), *Neisseria* (0.9850%), *Streptococcus* (0.9831%), *Veillonella* (0.9726%), *Mycolicibacter* (0.1964%), *Primate bocaparvovirus 1* (0.1689%), *Capnocytophag* (0.1244%), and *Actinomyces* (0.1100%) (Table 4). Although the rank of fungi were high on the list, there were no significant differences between the reads of fungi (23.75%) and non-fungi (22.59%) (non-parametric tests, P > 0.05). The distribution of pathogens was compared between the two groups. From the 27 pathogens discovered, six pathogens were evenly distributed in the two groups, including MTBC, *Prevotella*, *Haemophilus*, *Capnocytophag*, *Streptococcus*, *Actinomyces*.

**Table 4:** The distribution of microorganisms at the genera level that were found in the samples of PTB patients

Genera	Reads	α* (%)	β*	γ*	Z-score
MTBC	81,988	53.6623	36/92	36/36	4.9093
*Candida*	13,731	8.9871	3/92	1/36	0.5308
*Aspergillus*	11,596	7.5898	3/92	3/36	0.3938
*Nakaseomyces*	10,961	7.1741	1/92	1/36	0.3531
*Human gammaherpesvirus 4*	10,727	7.0210	3/92	3/36	0.3381
*Mycoplasma*	7772	5.0869	2/92	2/36	0.1485
*Prevotella*	6241	4.0848	5/92	5/36	0.0503
*Haemophilus*	2340	1.5316	4/92	4/36	−0.1999
*Human alphaherpesvirus 1*	1784	1.1677	2/92	2/36	−0.2356
*Neisseria*	1505	0.9850	3/92	3/36	−0.2535
*Streptococcus*	1502	0.9831	4/92	4/36	−0.2537
*Veillonella*	1486	0.9726	1/92	1/36	−0.2547
*Mycolicibacter*	300	0.1964	1/92	1/36	−0.3308
*Primate bocaparvovirus 1*	258	0.1689	1/92	1/36	−0.3335
*Capnocytophag*	190	0.1244	2/92	2/36	−0.3378
*Actinomyces*	168	0.1100	4/92	4/36	−0.3393
*Human betaherpesvirus 5*	79	0.0517	3/92	3/36	−0.345
*Human betaherpesvirus 6B*	58	0.0380	1/92	1/36	−0.3463
*Pneumocystis*	30	0.0196	3/92	3/36	−0.3481
*Staphylococcus*	23	0.0151	1/92	1/36	−0.3486
*Torque teno virus*	15	0.0098	2/92	2/36	−0.3491
*Cardiobacteriu*	11	0.0071	1/92	1/36	−0.3493
*Acinetobacter*	11	0.0071	2/92	1/36	−0.3493
*Torque teno virus 16*	4	0.0026	1/92	1/36	−0.3498
*Rothia*	3	0.0020	1/92	1/36	−0.3498
*Klebsiella*	1	0.00060	1/92	1/36	−0.3500
*Hepatitis B virus*	1	0.00060	1/92	1/36	−0.3500

*In the column of α, β and γ refer to the ratio of each pathogen sequenced read in total read, frequency of each pathogen, and patients of each pathogen

Additionally, we found that only 11 of the 36 cases had results of the specific *Mycobacterium* strain. They, respectively, were *Mycobacterium tuberculosis* (10/36, 27.78%) and *Mycobacterium microtus* (1/36, 2.78%). The most frequent patterns of mixed microbial composition were bacteria and virus mixed (5/36, 13.89%), followed by bacteria and fungus mixed (3/36, 8.33%) and mixture of bacteria, fungi and viruses (3/36, 8.33%). No significant between-group differences were found (multiple comparisions, *P* > 0.05).

### Potential usage in patients with viral identification

In order the sequencing reads of the 8 viruses from 9 patients were *Human gammaherpesvirus 4* (EBV) (n = 3, reads = 10,727), followed by *Human alphaherpesvirus 1* (n = 2, reads = 1784), *Primate bocaparvovirus 1* (n = 1, reads = 258), Human *betaherpesvirus 5 *(CMV) (n = 3, reads = 79), *Human betaherpesvirus 6B* (n = 1, reads = 58)*, Torque teno virus* (n = 2, reads = 15)*, Torque teno virus 16*(n = 1, reads = 4) *and Hepatitis B virus* (n = 1, reads = 1) (Table 4). Combined with the results of T cell subpopulation test, nearly half of the patients (4/9, patient NO.14, 15, 21, 31) were considered immunodeficiency hosts and may have been diagnosed with a chronic viral infection or hospital-acquired infections. All of them did not receive antiviral medication before the mNGS test. Additionally, there was a quite high percentage of patients (8/9, 88.89%) wide-spectrum antibiotics, including levofloxacin, moxifloxacin, and cefixime, while 7 of 9 patients were suspected of inappropriate antibiotic usage.

## Discussion

This is a single-center retrospective study to assess the diagnostic performance of BALF and lung biopsy tissue for mNGS detecting MTBC. Previous studies have shown the value of mNGS on MTBC detection, most of which generally involved BALF and sputum samples. This is the first study to directly compare BALF with lung biopsy tissue on the use of mNGS in MTBC detection. Preceding studies have indicated that the efficacy of culture, Xpert, and mNGS was significantly affected by anti-TB treatment [18]. All enrolled patients in our research had not previously received anti-TB treatment that ensures the objectiveness and accuracy of the research results. Moreover, we implemented the R-EBUS probe lesions suspected of PTB location when TBLB to make certain of higher diagnostic rates of mNGS from lung biopsy tissues.

The keys to managing infectious diseases include the following vital aspects (1) control the source of infection, (2) cut off the route of transmission, and (3) protect the susceptible population [19]. Early bacteriological diagnosis of PTB case is crucial to limit the spread of infections [20]. Nevertheless, early detection of PTB from other lung infections is difficult by traditional methods, particularly for patients with mild symptoms or uncharacteristic appearances on imaging [21, 22]. In 2020, the incidence and mortality rates of PTB were 47.7644 per 100,000 and 0.1367 per 100,000 in China. The positive tuberculosis case detection rate (CDR) is less than 40% in China, which would place a heavy burden on health care services, and carry enormous consequences for societies and economies [23]. Therefore, it is critical in improving the diagnosis of patients with PTB. Recently, the popularity of mNGS has drawn extensive concerns, particularly with respect to shortening turnaround time and guaranteeing accurate pathogen detection for diagnosis [8]. Xpert is WHO-recommended rapid test that simultaneously detect tuberculosis and rifampicin resistance in people with signs and symptoms of tuberculosis. This assay has been proven useful to diagnose PTB, with 89% sensitivity and 99% specificity [24]. Previous research has shown that Xpert performs similar detection efficiency to mNGS in MTBC detection [18]. In the Health Industry Standards of the People's Republic of China—Tuberculosis Diagnosis issued in 2017 [25], positive Xpert test of MTBC and lung imaging examination were included in the diagnostic standards of tuberculosis, which is highly recognized for the application value of the Xpert test in tuberculosis diagnosis. Removing false-positive results is a primary challenge for mNGS analysis. Therefore, for the patients detected MTBC positive by mNGS, we had implemented Xpert as part of the validation process. In our study, mNGS and Xpert displayed similar overall MTBC detection efficiency, which was also much superior to AFB (Pearson Chi-Square test, < 0.05), which demonstrates that mNGS is a reliable tool for MTBC detection, whether using BALF or lung biopsy specimens. However, during the process of clinical diagnosis and treatment, we noticed that major deficiencies of Xpert are as follows. Firstly, according to the WHO report, it has not improved global case detection rates and shows limited efficacy in extrapulmonary TB (ETB) [26]. Secondly, The Xpert and PCR laboratory tests are DNA tests of MTBC used to diagnose tuberculosis infection, but they cannot diagnose NTM infection [27]. Thirdly, the detection limit of Xpert is 131 CFU/ml; any unit lower than this value cannot be detected, resulting in negative results. Moreover, although Xpert shows both high sensitivity and specificity, and suggests its high value in PTB diagnosis; however, the application of pleural fluid is still limited, and should be improved [28]. Therefore, in some special cases, mNGS can be recognized as an important complement to Xpert in tuberculosis diagnosis. Both methods have their own pros and cons that seem to complement each other. For patients with complex disease, the combining mNGS and Xpert to diagnose PTB can improve the diagnostic rate [29].

Sputum specimens are mostly used as respiratory samples due to advantages such as convenience and security. However, it inevitably exists potential pollution from the oral cavity microbiota and upper respiratory tract (URT). BAL is playing an increasingly important role in the diagnosis of respiratory diseases because it leads in the fields of accountability, practicality and safety for operation [30, 31]. When inflammation lesions are anatomically limited to the surrounding lung parenchyma, the most accessible site should be biopsied. TB, an intracellular bacterium, tends to release fewer extracellular nucleic acids due to its intracellular growth characteristics, which make it difficult to detect [32]. However, the sensitivity for detecting TB will be improved since biopsy may lead to the destruction of tissues and cells. In our research, we found that mNGS samples from BALF and lung biopsy tissue varied in detecting MTBC. Lung biopsy tissue mNGS was more sensitive than BALF to the MTBC detection. Lung biopsy is indeed more invasive in patients than in BALF. Therefore, we should weigh the pros and cons carefully when we can gain the same benefit from both options. For patients with PPLs or who have contraindications of BAL procedures, detection of MTBC using biopsy tissue by mNGS is a more appropriate diagnostic method. Additionally, the sampling method of alveolar lavage fluid is single, while lung biopsy tissue can be obtained by R-EBUS-TBLB, percutaneous lung puncture biopsy (PNLB) and thoracoscopic biopsy. According to clinical observation, most patients have poor tolerance for BAL, particularly for the elderly patients complicated with cardiovascular and cerebrovascular diseases who are rejected for painless tracheoscopy when the risk of anesthesia is high. The pain and discomfort caused by ordinary bronchoscopy is so unbearable that those patients with a specific location of lung lesions prefer to choose PNLB. Besides, thoracoscopic examination for patients with suspected tuberculous pleurisy can accurately determine the lesion site and identify the lesion. If the examination results from thoracoscopic sampling specimens are positive, tuberculous pleurisy can be clearly diagnosed. However, the lung biopsy samples by R-EBUS-TBLB and PNLB are usually small and thus it is not enough to be used for MTBC culture after mNGS and pathological examination. Paraffin-embedded tissue (FFPE) can be used for acid-fast staining identification, but cannot be applied to culture. Tissue-mNGS detection has a unique application value in the abovementioned context. When it comes to the situations that BALF samples have limited value to catch pathogens for special lesion sites (such as PPLs) or the patients have contraindications to BAL procedures, lung biopsy tissue is an optional specimen for MTBC detection by mNGS. MTBC was considered positive when no less than 1 read was mapped to either the species or genus level because of the low yields of DNA extraction and rare specimen contamination. [14, 15]. NGS tends to detect all nucleotide sequences not only from the samples but also those acquired from the contamination, which remains a huge challenge on interpreting of mNGS results. One of the controversial subjects is that the cut-off values for low MTBC sequencing reads by mNGS may indicate a false-positive result. Our study spotted 6 cases with one read mapping while reads of the other 6 cases were less than ten, but these 12 cases were clinically confirmed by successfully responsive to anti-TB treatment. This research revealed that mNGS shows unique advantages and approaches a specificity of 100% in the diagnosis of PTB with a low MTBC burden.

Significantly, although mNGS possesses the ability to identify MTBC and NTM, because of the high internal homology and relatively insufficient coverage, the species within the MTBC was difficult to determine. A total of 11 cases had specific PTB strains among the 36 patients in our research. Based on accumulating evidence, making treatment options tends to depend on different strains. Therefore, it is necessary to explore the future potential of identifying the species MTB. Analogous limitations had also affected the analysis of other pathogens. Species-level are usually not sufficiently precise for characterization as the DNA chains for mNGS DNA sequencing are too short to offer enough information for identification. Therefore, according to the inhomogeneous data on sequencing reads of each pathogen in our study, the pathogens were discussed only at the genera-level while the further research is needed to reveal the inner layer details in species-level of pathogens.

Chronic viral infections can induce inflammatory reactions and suppress processes that drive immune defenses to enhance viral elimination, which increases the severity of MTBC coinfection [33]. MTBC coinfection has been linked to pulmonary *Kaposi's sarcoma-associated* *herpesvirus* (KSHV) infections [34]. CMV, EBV and other viruses tend to be detected in the BALF of many patients who are affected by pulmonary infection after kidney transplantation [35]. We found 9 cases complicated with viral infection. Detection of viral DNA or RNA by RT-qPCR remains a gold standard for the identification of infected patients. Unfortunately, independent validation experiments were not carried out in this study owing to this being a retrospective study. Therefore, a diagnosis of co-infection cannot be confirmed. Whether it is necessary to provide antiviral therapy according to a few reads of viruses remains uncertain. However, dynamic variations in the sequencing of reads and relative abundance from mNGS detection before and after treatment could indirectly reflect therapeutic effectiveness and determine whether these viruses were detected as infection or colonization [36]. Hence, more research is needed to affirm the reasonable cut-off values, which are vital elements of therapeutic options.

In our study, the lung virome and mycobiota were analyzed to confirm if they were involved in the alveolar environment. Our study found that the sequence of the microbiota such as *Human gammaherpesvirus 4*, *Human alphaherpesvirus 1*, *Human betaherpesvirus 5*, *Human betaherpesvirus 6B*, *Primate bocaparvovirus 1*, *Torque teno virus*, *Torque teno virus 16*, *Hepatitis B virus*, *Candida*, *Aspergillus*, *Nakaseomyces*, and *Pneumocystis* was detected. We attempted to analyze the relationships and differences among the 27 pathogens detected by mNGS. However, statistical analysis revealed that there was no significant difference among them. Further research is needed to determine the cut-off value of specific pathogens when identifying co-infecting. The detection of some genera that might have a few pathogenic species does not mean that there is a co-infection. More than that, it should confirm the pathogens using complementary methods such as qPCR as mNGS has some challenges in classification and identification. However, mNGS has potential applications in viral identification.

MTBCs can invade the vulnerable groups via the respiratory tract, digestive tract, or broken skin. A study showed that patients with TB had a marked change in bacterial community, then a disappearance of highly homogenous bacterial flora. From our awareness of TB pathology, mycobacteria invasion by injuring the defense of mucosal barrier function in the lower respiratory tract (LRT) is a crucial factor, facilitating the bacterial colonization [37, 38]. However, previous studies merely demonstrated the interaction between MTBC infection and microbiota [39]. The LRT was initially thought to be sterile despite its constant exposure to the environment [40]. However, it has recently been shown to not be a sterile state in the normal LRT with further relevant research [41]. It has been a hotspot in research area to the impact of tuberculosis and anti-tuberculosis therapy on the composition and modification of human lung microbial communities. However, the relationship between MTBC pulmonary infection and the resident lung microbial composition remains unclear. One study found that the abundance of the *Mycobacterium* and *Porphyromonas* genera was increased in TB lesions, while the *Cupriavidus* genus was reported as dominant and specific in patients with TB [37]. A study showed the difference in bacterial quantity from the URT and LRT, but no difference in composition [41]. Evidence from the other study suggested a significant reduction in alveolar microbiota diversity in BALF samples from patients with TB and interstitial pneumonia, compared to healthy volunteers who took part in the study, which was characterized by a common reduction in *Streptococcus* and *Acinetobacte* genus abundances for the two diseases, respectively [42]. It makes sense to investigate the role and diversity of the microbiome and metagenome in mediating MTBC infection. Once the correlation between MTBC infection and microbiota could be established, it will achieve a significant breakthrough in TB research.

The relatively high expense of the mNGS routine (3600 China Yuan, approach 570 dollars) restricts its extensive application and secondary test. Moreover, patients diagnosed with PTB previously were reluctant to receive a second test, which makes it difficult to the supervision and management of drug-resistant MTBC. Few reports were described the alveolar microbiota in BAL samples from patients with TB after anti-TB treatment. More patients will benefit from mNGS when the technique procedure is optimized and the cost is reduced.

This study has several limitations as follows. Firstly, our study involved DNA-based mNGS only; thus, only DNA was sequenced in the study, which means that all RNA viruses were excluded including *Influenza virus*, *Bunyavirus*, *Hantavirus* and so on. Secondly, considering the medical expenses, the enrolled patients did not receive both mNGS analysis of BALF and lung biopsy tissue at the same time. Thirdly, there were some barriers to follow-up patients with PTB because they were required to undergo further treatment in a specialist hospital. Finally, this was a single-center retrospective study. A multicenter, prospective cohort study should be performed in the future.

## Conclusions

In conclusion, this study evaluated the performance of BALF-based or lung biopsy tissue-based mNGS for microbiologic diagnosis of MTBC. Usually, qualified BALF is an ideal sample for mNGS detection of MTBC. When it comes to the situations that BALF samples have limited value to catch pathogens for special lesion sites or the patients have contraindications to BAL procedures, lung biopsy tissue is an optional specimen for MTBC detection by mNGS. However, whether lung tissue-mNGS is superior to BALF-mNGS in patients with MTBC infection requires further prospective multicenter randomized controlled studies with more cases.

## Data Availability

The mNGS sequence data presented in this study can be found online at: https://www.ncbi.nlm.nih.gov/Traces/study/?acc=PRJNA784757. The datasets used and/or analyzed during the current study are available from the corresponding author on reasonable request.

## Abbreviations

TB: Tuberculosis
PTB: Pulmonary tuberculosis
MTBC: *Mycobacterium tuberculosis complex*
mNGS: Metagenomic next-generation sequencing
BALF: Bronchoalveolar lavage fluid
TBLB: Transbronchial lung biopsy
PNLB: Percutaneous lung puncture biopsy
SARS: Severe acute respiratory syndrome
COVID-19: Coronavirus disease 2019
AFB: Acid-fast bacillus
WHO: World Health Organization
Xpert: GeneXpert MTB/RIF
CT: Computerized tomography
PPD: Purified protein derivative
BAL: Bronchoalveolar lavage
CPLP: CT-guide percutaneous lung puncture
R-EBUS-TBLB: Radial endobronchial ultrasound transbronchial lung biopsy
PPLs: Peripheral pulmonary lesions
DNA: Deoxyribonucleic acid
PCR: Polymerase chain reaction
DNB: Deoxyribonucleic acid nanoball
NTM: Nontuberculous mycobacteria
CI: Confidence intervals
PPV: Positive predictive value
NPV: Negative predictive value
EBV: *Human gammaherpesvirus 4*
CMV: *Human betaherpesvirus 5*
CDR: Tuberculosis case detection rate
ETB: Extrapulmonary tuberculosis
KSHV: *Kaposi's sarcoma-associated herpesvirus*
URT: Upper respiratory tract
LRT: Lower respiratory tract
